# Circulating Extracellular Vesicles and Their miR “Barcode” Differentiate Alcohol Drinkers With Liver Injury and Those Without Liver Injury in Severe Trauma Patients

**DOI:** 10.3389/fmed.2019.00030

**Published:** 2019-02-25

**Authors:** Akiko Eguchi, Niklas Franz, Yoshinao Kobayashi, Motoh Iwasa, Nils Wagner, Frank Hildebrand, Yoshiyuki Takei, Ingo Marzi, Borna Relja

**Affiliations:** ^1^Department of Gastroenterology and Hepatology, Mie University Graduate School of Medicine, Tsu, Japan; ^2^JST, PRESTO, Saitama, Japan; ^3^Department of Trauma, Hand and Reconstructive Surgery, University Hospital Frankfurt, Goethe University, Frankfurt, Germany; ^4^Department of Orthopaedic Trauma, RWTH Aachen University, Aachen, Germany

**Keywords:** outcome, inflammation, miR, extracellular vesicles, alcohol, trauma

## Abstract

**Short Summary:** Extracellular vesicles (EVs), released during tissue/cell injury, contain a “barcode” indicating specific microRNAs (miRs) that can uncover their origin. We examined whether systemic EVs possessing hepatic miR-signatures would indicate ongoing liver injury and clinical complications in trauma patients (TP). We grouped the patients of alcoholic drinkers into “alcohol-drinkers with liver injury (LI)” (EtOH with LI) or “alcohol-drinkers without LI” (EtOH w/o LI) and we compared these groups to “non-drinkers” (no EtOH). When we examined patient blood from the EtOH with LI group we found the total number of EVs to be increased, along with an increase in miR-122 and let7f—two EV-associated miRNAs—and several inflammation-associating cytokines, such as interleukin (IL)-6 and IL-33. In contrast, all of the aforementioned readouts were found to be decreased in the EtOH w/o LI group. These novel data demonstrate that hepatocyte damage in alcohol-intoxicated trauma patients presenting with liver injury can be reflected by an increase in circulating serum EVs, their specific miR-“barcode” and the concomitant increase of systemic inflammatory markers IL-6 and IL-33. Anti-inflammatory effect of alcohol-drinking in EtOH w/o LI can be presented by a reduced number of hepato-derived EVs, no upregulation of IL-6 and IL-33, and a miR “barcode” different from patients presenting with liver injury.

**Background:** Alcohol abuse is associated with (neuro)protective effects related to (head) injuries, and with negative effects regarding infection rates and survival in severely injured trauma patients (TP). Extracellular vesicles (EVs), which are released during tissue and/or cell injury, can contain a “barcode” including specific microRNAs (miRs) that uncover their origin. We examined whether EVs with a hepatic miR signature can be systemically measured, and whether they can indicate ongoing liver injury in alcohol-intoxicated TP and foretell clinical complications.

**Patients/Methods:** We enrolled 35 TP and measured blood EVs, IL-6, TNF-alpha, IL-1beta, IL-10 and IL-33, alcohol (ethanol, EtOH) concentration (BAC), GLDH, GGT, AST, ALT, leukocytes, platelets, and bilirubin. Within circulating EVs we measured the expression levels of miR-122, let7f, miR21, miR29a, miR-155, and miR-146a. Patients of alcohol-drinkers were grouped into “alcohol drinkers with liver injury (LI)” (EtOH with LI) or “alcohol drinkers without LI” (EtOH w/o LI) and compared to “non-drinkers” (no EtOH). We assessed systemic injury characteristics and the outcome of hospitalization with regard to sepsis, septic shock, pneumonia, or mortality.

**Results:** EtOH with LI patients had significantly increased rates of pneumonia *vs*. the EtOH w/o LI group. EVs, IL-6, and IL-33 levels were significantly increased in EtOH with LI vs. EtOH w/o LI group (*p* < 0.05). EV number correlated positively with ALT and IL-6 (*p* < 0.0001). Two miRs, miR-122 and let7f, were increased only in the blood EVs from the EtOH with LI group (*p* < 0.05). Five miRs, miR-122, let7f, miR-21, miR-29a, and miR-146a, were reduced in the blood EVs from the EtOH w/o LI group, vs. no EtOH (*p* < 0.05). Notably miR-122 correlated significantly with increased bilirubin levels in the EtOH with LI group (*p* < 0.05).

**Conclusions:** Liver injury in alcohol-intoxicated TP is reflected by increased EV numbers, their specific miR barcode, and the correlated increase of systemic inflammatory markers IL-6 and IL-33. Interestingly, severely injured TP without liver injury were found to have a reduced number of liver-derived EVs, no observed inflammatory infiltration and reduced specific miR “barcode.”

## Introduction

Trauma is one of the most common causes of mortality in young patients worldwide (1). During their clinical course, patients frequently die from critical immune dysregulations (2). Despite extensive research on the post-traumatic inflammatory response, mortality rates remain high and the underlying pathophysiology is still not fully understood (3). Alcohol abuse is associated with nearly 50% of all admissions to emergency departments, moreover the immune-modulating effect of alcohol significantly impacts patient outcome (4–6). However, reports regarding the outcome rates in intoxicated patients remain inconclusive. Alcohol has been shown either to have no effects on the post-injury outcome, or to increase the risk for complications and mortality (5–9). Paradoxically, alcohol has been shown to provide a neuroprotective influence in traumatic brain injury and reduced incidence of pneumonia in patients (10, 11). In summary, the immune-suppressive effect of acute alcohol consumption may reduce inflammation (12–14), whereas continuous intoxication due to chronic alcoholism is often accompanied by severe liver injury and associated pro-inflammatory reactions, which can exacerbate post-injury complications. Thus, there is currently a great interest in developing reliable, non-invasive tools for the evaluation of pre-traumatic liver injury in alcohol-intoxicated trauma patients.

Extracellular vesicles (EVs) are small, membrane vesicles that are released from activated or dying cells. Presently there exists two distinct populations of EVs based on size: exosomes (30–100 nm) and microparticles (100–1000 nm). EVs carry the molecular signature of their origination cells including proteins, mRNAs, miRNAs (miRs), and lipids (15–18), which make EVs effective cell-to-cell communicators (19, 20). Specific EVs from activated or dying cells can be used as biomarkers. For example, circulating EVs with liver-specific proteome markers and miRs are released from lipotoxic hepatocytes in nonalcoholic fatty liver disease (21, 22). Recently, it has been shown that damaged hepatocytes from alcoholic liver disease (ALD) release a key source of EVs containing a specific miR “barcode” (23). Three miRNAs—let7f, miR-29a, and miR-340—were significantly increased in the blood EVs from mild alcoholic steatohepatitis (mASH) mice, but not in the blood EVs from other mouse models of chronic liver injury, including bile duct ligation, obesity, and nonalcoholic steatohepatitis, as well as EVs released from hepatocytes exposed to ethanol (23). Moreover, let7f, miRNA-29a, and miR-340 were specifically enhanced in not only the mASH model, but human ALD as well (23). Eguchi et al. and others have summarized that injured hepatic cells are the major source of EVs, and those EVs contain a specific miR “barcode,” which is detectable in the blood (21, 23, 24). Notably, the expression of liver-specific miR-122 was increased by chronic ethanol feeding (25). miR-21 was closely associated with fibrotic liver disease *in vivo*, and therefore proposed as a potential plasma biomarker for hepatic fibrosis (26). On the other hand, miR-146a plays a key role in controlling inflammation by inhibiting the inflammatory response in macrophages, monocytes, and keratinocytes (27–29). To determine whether the systemic EV population from alcohol-intoxicated trauma patients with and without liver injury could be strictly characterized by its miR composition, the expression patterns of miR-122, let7f, miR-21, miR-29a, miR-155, and miR-146a were analyzed.

Here, we tested the hypothesis that the condition of pre-existing liver injury in alcohol-intoxicated patients will significantly affect trauma patient outcome and will be reflected by EV release, with its injury-related hepatocyte-specific miR “barcode,” and the potential for subsequent inflammatory changes.

## Patients and Methods

### Ethics

Patients and 13 healthy volunteers were included in the University Hospital of the Goethe-University Frankfurt with institutional ethics committee approval in accordance with the Declaration of Helsinki and following STROBE-guidelines (30). Written informed consent was obtained from all enrolled subjects in accordance with ethical standards. All enrolled subjects signed the informed consent forms themselves or informed consent was obtained from the nominated legally authorized representative consented on the behalf of participants as approved by the ethical committee.

### Patients

Thirty-five patients with a history of acute trauma with an Injury Severity Score (ISS) ≥16 between 18 and 80 years of age were included. All patients with known pre-existing immunological disorders, immunosuppressive and anti-coagulant medication, burns, concomitant acute myocardial infarction, or thromboembolic events were excluded. All patients were negative for both hepatitis B virus surface antigen and antibody to hepatitis C virus. Serum blood alcohol concentration (BAC) was measured immediately on admission to the emergency department. The definition of liver injury was performed based on the rise in serum levels of gamma-glutamyl transpeptidase (GGT), aspartate aminotransferase (AST), alanine aminotransferase (ALT), and affirmed by bilirubin above baseline, which were diagnosed with every admission. Glutamate dehydrogenase (GLDH) was determined as well. Since most causes of liver cell injury are associated with a greater increase in ALT than AST; an AST to ALT ratio of 2:1 or greater is suggestive of alcoholic liver disease, particularly in the setting of an elevated GGT (31, 32). The evaluation of the AST to ALT ratio and GGT levels have been analyzed at the admission day and during the hospital stay daily for 10 post-injury days. Based on the data obtained from other studies, the upper normal limits for ALT and AST were set at 40 U/L and 50 U/L for GGT, respectively (33–35). However, to better distinguish liver injury from other etiology, we applied the increased AST:ALT >2, and increase in GGT >50 U/L during the hospital stay (36, 37). The combined elevation was used for the group allocation. Additionally, as described above all patients with other causes of liver disease such as hepatitis B or C, which potentially could have increased levels of the applied biomarkers were excluded based on laboratory virology/serology. Furthermore, to confirm the correct group allocation, we have determined IL-33 (as a damage-associated molecular pattern, DAMP, alarmin) in samples obtained at admission of patients to the emergency department. Plasma levels of IL-33 were elevated specifically in ALD patients (38). Patients were grouped to “alcohol drinkers with liver injury (LI)” (EtOH with LI) and “alcohol drinkers without LI” (EtOH w/o LI) vs. “non-drinkers” (no EtOH).

### Study Setting

All trauma patients were treated upon admission according to the Advanced Trauma Life Support (ATLS) standards and the polytrauma guidelines (39, 40). Trauma mechanism and the demographic data (age and gender), injury severity parameters (ISS, AIS head, chest, abdomen, extremities), ICU and in-hospital length of stay as well as in-hospital mortality were registered. The injury severity from trauma was calculated using the ISS based on the Abbreviated Injury Scale (AIS) score (41, 42). Sepsis was diagnosed according to sepsis-3 criteria (43). Briefly, sepsis was defined as life-threatening organ dysfunction caused by a dysregulated host response to infection. Organ dysfunction can be represented by an increase in the Sequential [Sepsis-related] Organ Failure Assessment (SOFA) score of 2 points or more (43, 44). Septic shock was clinically identified by a vasopressor requirement to maintain a mean arterial pressure of 65 mm Hg or greater and serum lactate level greater than 2 mmol/L (>18 mg/dL) in the absence of hypovolemia (43, 45). Pneumonia development during the post-traumatic clinical course was defined by radiologic, clinical and bacteriologic findings with the presence of new pulmonary infiltrates on chest X-ray and at least one of the following criteria: positive blood culture, bronchial alveolar lavage and/or sputum culture (46).

### Blood Processing and Analysis

Blood samples were obtained from severely traumatized patients on admission to the emergency department for routine diagnostics or for laboratory investigations. The routine diagnostic was followed up daily until post-injury day 10. Blood samples were obtained as early as possible after admission of the patient in pre-chilled ethylenediaminetetraacetic acid (EDTA) tubes (BD vacutainer, Becton Dickinson Diagnostics, Aalst, Belgium) and kept on ice. Blood was centrifuged at 2000 × g for 15 min at 4°C and the supernatant was stored at −80°C until analysis.

Cytokine concentrations were measured by IL-6 Eli-pair ELISA-Assay, and IL-10 Eli-pair ELISA-Assay (Diaclone, Hoelzel Diagnostica, Cologne, Germany), human IL-33, human IL-1beta/IL-1F2 and human TNF-alpha DuoSet ELISA (R&D Systems) according to manufacturer's instructions. Blood counts (leukocytes and platelets) were obtained by standard clinical methods using the Sysmex XE-2100 automated blood cell counter (Sysmex Europe GmbH, Norderstedt, Germany). Alcohol concentration was determined using the diagnostic set serum ethanol by Cobas 8000 Modular Analyzer (both Roche Diagnostic, Mannheim, Germany).

### Analysis of Extracellular Vesicles

Circulating EVs in plasma were analyzed as previously described (23). Briefly, circulating EVs were stained with final 4 μg/mL of calcein-AM (Invitrogen, San Diego, CA) at least 30 min in dark at room temperature. The number of EVs was determined using 2.5-μm UV-conjugated Alignflow alignment beads (Life Technologies) as the size standards for flow cytometry (BD Cant II; BD Biosciences, San Jose, CA), and data were analyzed using FlowJo software (TreeStar, Ashland, OR). Before measurement of samples, gate was confirmed using several differently sized beads [Spherotech nano fluorescent particle size standard kit (Spherotech, Lake Forest, IL) and FluoSpheres biotin-labeled 0.04 μm yellow-green (Life Technologies)].

### Protein Abundance and miR Levels of Isolated EVs

Circulating EVs were isolated *via* qEV (Izon Science, Cambridge, MA) according to manufacturer's instruction. Briefly, plasma was applied on the qEV column and fractions 6–10 were collected. EV fractions were concentrated with Amicon Ultracel-3K (EMD Milllipore, Temecular, CA). For protein abundance, isolated EVs were resolved in TGX^TM^ precast gels and transferred to nitrocellulose membrane (BioRad, Hercules, CA). Blotted membranes were incubated with blocking reagent and primary antibody, anti-CD9 (BioLegend, San Diego, CA), in Can Get solution (TOYOBO, OSAKA, Japan) followed by peroxidase-conjugated secondary antibody incubation (GE Healthcare Life Sciences, Pittsburgh, PA). The membrane was treated with azide-TBST to remove HRP. Protein bands were visualized using enhanced chemiluminescence reagents (Thermo Fisher Scientific, Waltham, MA) and digitized using a charge-coupled device camera (LAS4000 mini; Fuji Film, Tokyo, Japan). Expression intensity was quantified by Multi Gauge software (Fuji). For miR levels, encapsulated miRNAs were extracted from purified EVs *via* qEV column using miRNase (Quiagen) according to the manufacturer's instruction. The templates were made from 10 ng of total RNA using TaqMan advanced miRNA cDNA synthesis kit (Life technologies). Real-time PCR quantification for miRNA expression was performed using a TaqMan advanced miRNA assay (Life Technologies). Cq value was converted to relative number using power formulation.

### Statistics

Kruskal-Wallis test with a Dunn‘s *post-hoc* test was used. Chi-square test was applied for the analyses of proportions. Correlation analysis was done using Pearson's test analysis. All data were tested for normal distribution by Kolmogorov-Smirnov test with Dallal-Wilkinson-Lilliefor correction. Data are presented as the mean ± standard deviation (SD) unless otherwise stated. A *p*-value < 0.05 was considered statistically significant. GraphPad Prism 6.0 software (GraphPad Software Inc. San Diego, CA) was used to perform the statistical analysis.

## Results

### Study Cohort and Characteristics

**Figure 1:**
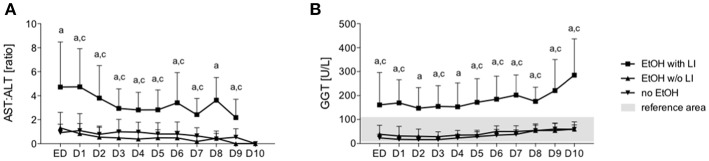
Ratio of aspartate aminotransferase (AST) to alanine aminotransferase (ALT) and levels of gamma-glutamyl transpeptidase (GGT) in trauma patients. Upon admission of severely injured trauma patients to the emergency department (ED) up to 10 days after admission, AST, ALT, and GGT were measured. Ratio of AST to ALT is shown **(A)** and GGT levels **(B)**. EtOH, ethanol; EtOH with LI, alcohol drinkers with liver injury; EtOH w/o LI, alcohol drinkers without liver injury; no EtOH, non-drinkers. Data are presented as mean ± SD. ^*^p < 0.05 in a, EtOH with LI vs. no EtOH; b, EtOH w/o LI vs. no EtOH; and c, EtOH with LI vs. EtOH w/o LI.

A total of 30 male and 5 female patients admitted to the emergency department with trauma met the inclusion criteria and were considered for our cohort. The mean age was 38.89 ± 2.69 and all patients were seriously injured (ISS: 26.76 ± 1.62). Patient injury location broke down as follows: 16 AIS head ≥3, 20 AIS chest ≥3, 5 AIS abdomen ≥3 and 7 AIS extremity ≥3. Of these patients, six were categorized as the EtOH with LI group and 14 were included in the EtOH w/o LI group. Upon admission to the emergency department, serum levels of GGT, AST, and ALT were significantly elevated in the EtOH with LI group when compared to the EtOH w/o LI or no EtOH groups (GGT: 161.3 ± 50.91, 38.63 ± 7.25 and 23.45 ± 4.70 U/L; AST: 288.0 ± 150.6, 73.64 ± 16.23 and 119.0 ± 26.81 U/L and ALT: 159.2 ± 61.69, 54.31 ± 7.09 and 96.18 ± 22.68 U/L in EtOH with LI, EtOH w/o LI and no EtOH, respectively; *p* < 0.05). Similar cohort data were also found for GLDH (GLDH: 32.68 ± 2.48, 8.96 ± 1.41 and 13.39 ± 4.44 U/L; *p* < 0.05). All patients within the EtOH with LI group had significantly increased AST: ALT ratio measurements >2 and a significant increase in GGT compared with the EtOH w/o LI or no EtOH groups during the hospital stay time period (*p* > 0.05, Figures 1A,B). BAC concentrations in the EtOH with LI and EtOH w/o LI groups were 1.61 ± 0.60 g/L (1.29 ± 0.49‰) and 2.09 ± 0.14 g/L (1.69 ± 0.11‰), respectively. A total of 15 low BAC patients were allocated to the no EtOH group, regardless of whether they fulfilled the criteria for liver injury. The no EtOH group had the lowest median age, however neither age nor gender were significant measures between the groups (Table 1). As a source of injury, falls were the most predominant trauma mechanism in both EtOH groups, especially when compared to the no EtOH group, however this difference was not statistically significant (Table 2). Traumatic head injuries were the most common injury pattern in the EtOH with LI (50.00%) and the EtOH w/o LI (64.29%) groups, but this incidence rate was not maintained in the no EtOH group (26.67%, Table 2). Liver injury was confirmed by significantly increased levels of serum IL-33 measured in samples obtained at admission from all patient groups, including healthy volunteers (EtOH with LI 431.4 ± 162.4 pg/mL; EtOH w/o LI 163.4 ± 66.26 pg/mL, no EtOH 68.08 ± 19.26 pg/mL; healthy volunteers 78.99 ± 38.13 pg/mL, *p* < 0.05). Serum levels of IL-1beta, TNF-alpha, and IL-10 were not statistically significant when compared between the groups.

**Table 1:** Summary of the study subjects.

**Subjects**	**All patients (*n* = 35)**	**EtOH with LI (*n* = 6)**	**EtOH w/o LI (*n* = 14)**	**no EtOH (*n* = 15)**	***p-*value**
Age (years), mean ± SD	38.89 ± 15.92	44.83 ± 14.39	41.79 ± 19.40	33.80 ± 11.88	a: 0.406 b: 0.791 c: >0.999
Sex (male, %)	30 (85.71%)	5 (83.33%)	12 (85.71%)	13 (86.67%)	a: 0.844 b: 0.941 c: 0.891
BAC (g/L)	1.15 ± 1.18	1.61 ± 1.47	2.09 ± 0.53	0.01 ± 0.01	a: 0.005 b: < 0.0001 c: >0.999
Per mille (‰)	0.93 ± 0.90	1.29 ± 1.19	1.69 ± 0.42	0.01 ± 0.01	a: 0.006 b: >0.0001 c: >0.999

*BAC, blood alcohol concentration; EtOH with LI, alcohol drinkers with liver injury; EtOH w/o LI, alcohol drinkers without liver injury; no EtOH, non-drinkers. Data are presented as mean ± SD unless stated otherwise. Exact p-value of a: EtOH with LI vs. no EtOH, b: EtOH w/o LI vs. no EtOH, and c: EtOH with LI vs. EtOH w/o LI*.

**Table 2:** Summary of trauma characteristics.

**Trauma characteristics**	**All patients (*n* = 35)**	**EtOH with LI (*n* = 6)**	**EtOH w/o LI (*n* = 14)**	**no EtOH (*n* = 15)**	***p-*value**
Trauma mechanism (falls)	20 (57.14%)	4 (66.67%)	9 (64.29%)	7 (46.67%)	a: 0.407 b: 0.340 c: 0.919
ISS	26.76 ± 9.46	29.50 ± 11.62	22.36 ± 5.10	28.79 ± 8.31	a: >0.999 b: 0.264 c: 0.671
**AIS ≥3 (*****n*****, %)**					
Head	16 (45.71%)	3 (50.00%)	9 (64.29%)	4 (26.67%)	a: 0.306 b: 0.042 c: 0.550
Chest	20 (57.14%)	3 (50.00%)	7 (50.00%)	10 (66.67%)	a: 0.477 b: 0.363 c: 1.000
Abdomen	5 (14.29%)	1 (16.67%)	0 (0%)	4 (26.67%)	a: 0.627 b: 0.037 c: 0.117
Extremity	7 (20.00%)	2 (33.33%)	1 (7.14%)	4 (26.67%)	a: 0.760 b: 0.164 c: 0.133

*AIS, abbreviated injury scale; EtOH with LI, alcohol drinkers with liver injury; EtOH w/o LI, alcohol drinkers without liver injury; ISS, injury severity score; no EtOH, non-drinkers. Data are presented as mean ± SD unless stated otherwise. Exact p-value of a: EtOH with LI vs. no EtOH, b: EtOH w/o LI vs. no EtOH, and c: EtOH with LI vs. EtOH w/o LI*.

### Outcome

Final patient outcomes and a listing of complications experienced by some in our cohort are depicted in Table 3. The length of stay in the ICU, or general admission in the hospital, was statistically comparable in all groups (Table 3). The development of sepsis on a per case basis was similar between the groups. The EtOH with LI group had significantly increased incidences of pneumonia when compared to the EtOH w/o LI group (*p* < 0.05, Table 3). The overall in-hospital mortality rate was statistically comparable among the groups, while the highest values were observed in the EtOH with LI (16.67%) group (Table 3).

**Table 3:** Summary of outcome variables.

**Outcome parameters**	**All patients (*n* = 35)**	**EtOH with LI (*n* = 6)**	**EtOH w/o LI (*n* = 14)**	**No EtOH (*n* = 15)**	***p-*value**
ICU stay (days)	8.82 ± 10.10	9.60 ± 6.91	5.93 ± 7.03	12.31 ± 13.15	a: >0.999 b: 0.311 c: 0.649
In-hospital stay (days)	20.74 ± 12.41	24.00 ± 17.86	17.71 ± 8.54	24.07 ± 12.69	a: >0.999 b: 0.629 c: >0.999
Sepsis (*n*, %)	8 (22.86%)	2 (33.33%)	1 (7.14%)	5 (33.33%)	a: 1.000 b: 0.082 c: 0.133
Septic shock (*n*, %)	1 (2.86%)	0 (0%)	0 (0%)	1 (6.67%)	a: 0.517 b: 0.326
Pneumonia (*n*, %)	9 (25.71%)	3 (50.00%)	1 (7.14%)	5 (33.33%)	a: 0.477 b: 0.082 c: 0.028
In-hospital mortality (*n*, %)	2 (5.71%)	1 (16.67%)	1 (7.14%)	0 (0%)	a: 0.105 b: 0.292 c: 0.515

*EtOH with LI, alcohol drinkers with liver injury; EtOH w/o LI, alcohol drinkers without liver injury; ICU, intensive care unit; no EtOH, non-drinkers. Data are presented as mean ± SD unless stated otherwise. Exact p-value of a: EtOH with LI vs. no EtOH, b: EtOH w/o LI vs. no EtOH, and c: EtOH with LI vs. EtOH w/o LI*.

### Reduced EV Numbers and Inflammation in Alcohol-Drinking Patients Without Liver Injury

**Figure 2:**
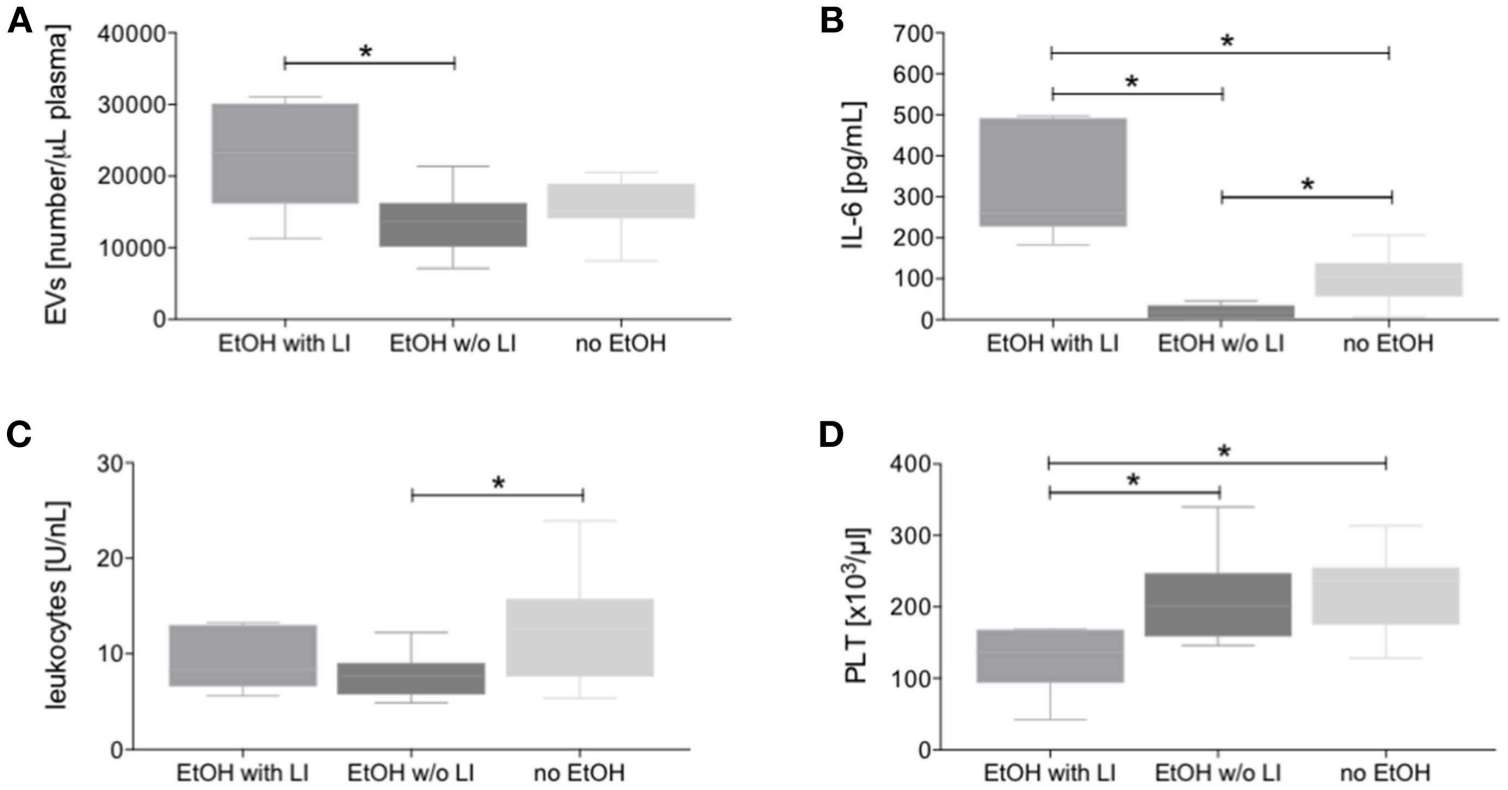
Circulating extracellular vesicles (EVs), interleukin (IL)-6, and cellular changes in blood from alcohol-intoxicated trauma patients. Dynamic light scattering flow analysis of blood EVs **(A)**, IL-6 levels **(B)**, total leukocytes **(C)**, and platelets (PLT, **D**) counts were determined upon admission of severely injured patients to the emergency department after trauma. EtOH, ethanol; EtOH with LI, alcohol drinkers with liver injury; EtOH w/o LI, alcohol drinkers without liver injury; no EtOH, non-drinkers. Data are presented as box and whiskers with 5–95 percentile. ^*^p < 0.05.

To investigate whether EVs are produced and released in trauma patients who have consumed alcohol and present with latent liver injury, blood EVs were measured and we observed that the EtOH with LI group showed a significant increase in EVs when compared to the EtOH w/o LI group (*p* < 0.05, Figure 2A).

In trauma patients, the EtOH with LI group displayed significantly increased systemic levels of the pro-inflammatory marker IL-6 when compared to the EtOH w/o LI or no EtOH groups, respectively (*p* < 0.05, Figure 2B). Interestingly, IL-6 levels were significantly decreased in the EtOH w/o LI group vs. both the EtOH with LI and the no EtOH patients, respectively (*p* < 0.05, Figure 2B). While IL-6 levels from the EtOH with LI and no EtOH groups were significantly increased compared to healthy volunteers, the EtOH w/o LI group did not statistically differ from healthy volunteers (EtOH with LI: 322.3 ± 135.0; EtOH w/o LI: 19.96 ± 15.44; no EtOH: 102.8 ± 63.46 and healthy volunteers: 1.02 ± 3.21 pg/mL). We also investigated whether the consumption of alcohol can influence leukocyte and platelet production. Total leukocyte numbers were decreased in both the EtOH with or w/o LI groups when compared to the no EtOH group (*p* < 0.05: EtOH w/o LI vs. no EtOH, Figure 2C), while platelet numbers were significantly decreased in the EtOH with LI group vs. the EtOH w/o LI and no EtOH groups, respectively (*p* < 0.05, Figure 2D).

**Figure 3:**
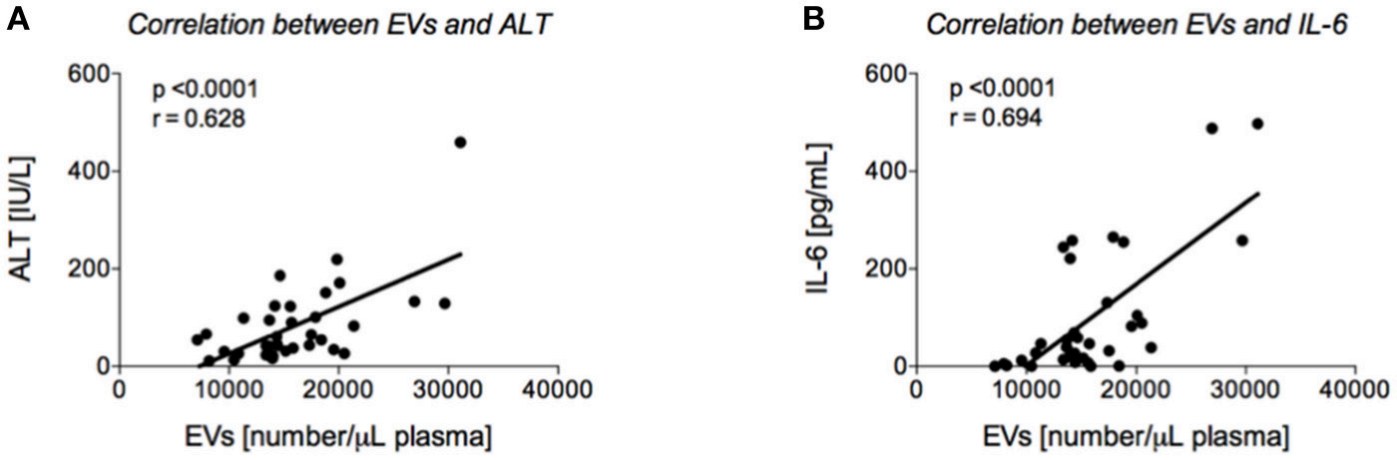
Correlation analyses between extracellular vesicles (EVs) and alanine aminotransferase (ALT) and interleukin (IL)-6, respectively. Positive correlation between EVs upon admission of severely injured patients to the emergency department (ED) and ALT **(A)** or IL-6 **(B)** values from ED is shown.

Our findings of an increased level of circulating EVs in the EtOH with LI traumatized patients led us to further examine the potential correlation between EVs and the degree of liver injury with or without concomitant inflammation. We were able to identify a positive correlation between EV number and liver damage as represented by patient ALT levels (*r* = 0.628, *p* < 0.05, Figure 3A). To uncover the impact of ongoing inflammation, EVs were correlated with systemic IL-6 levels, which showed a significant positive correlation between those two factors (*r* = 0.694, *p* < 0.05, Figure 3B).

### Circulating EVs in Blood From Alcohol-Intoxicated Trauma Patients Are Predominantly Derived From the Liver

**Figure 4:**
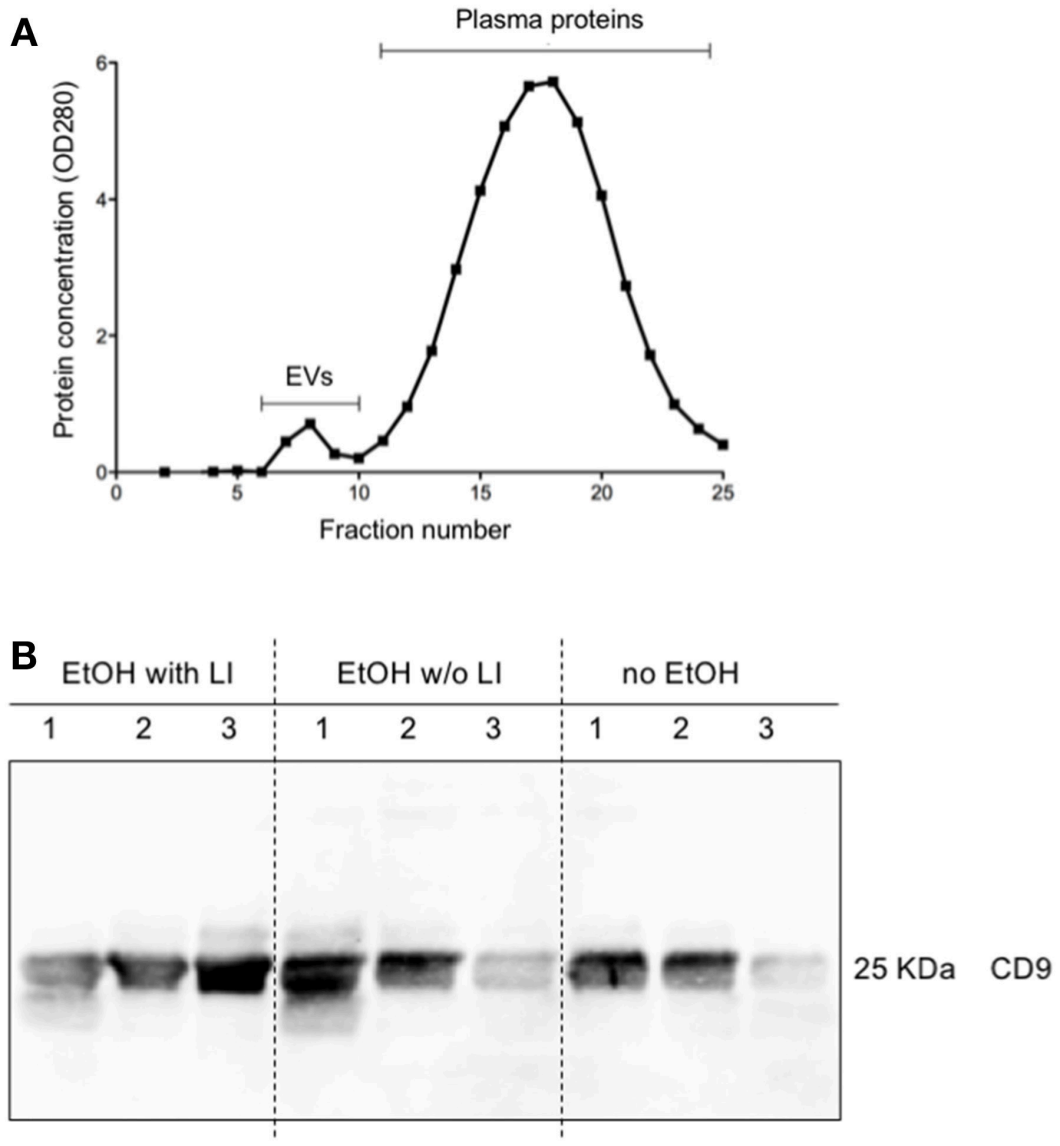
Characterization of extracellular vesicles (EVs). Isolation of circulating EVs *via* qEV column as a representative sample **(A)**. Protein expression of CD9 in isolated circulating EVs **(B)**. EtOH, ethanol; EtOH with LI, alcohol drinkers with liver injury; EtOH w/o LI, alcohol drinkers without liver injury; no EtOH, non-drinkers.

**Figure 5:**
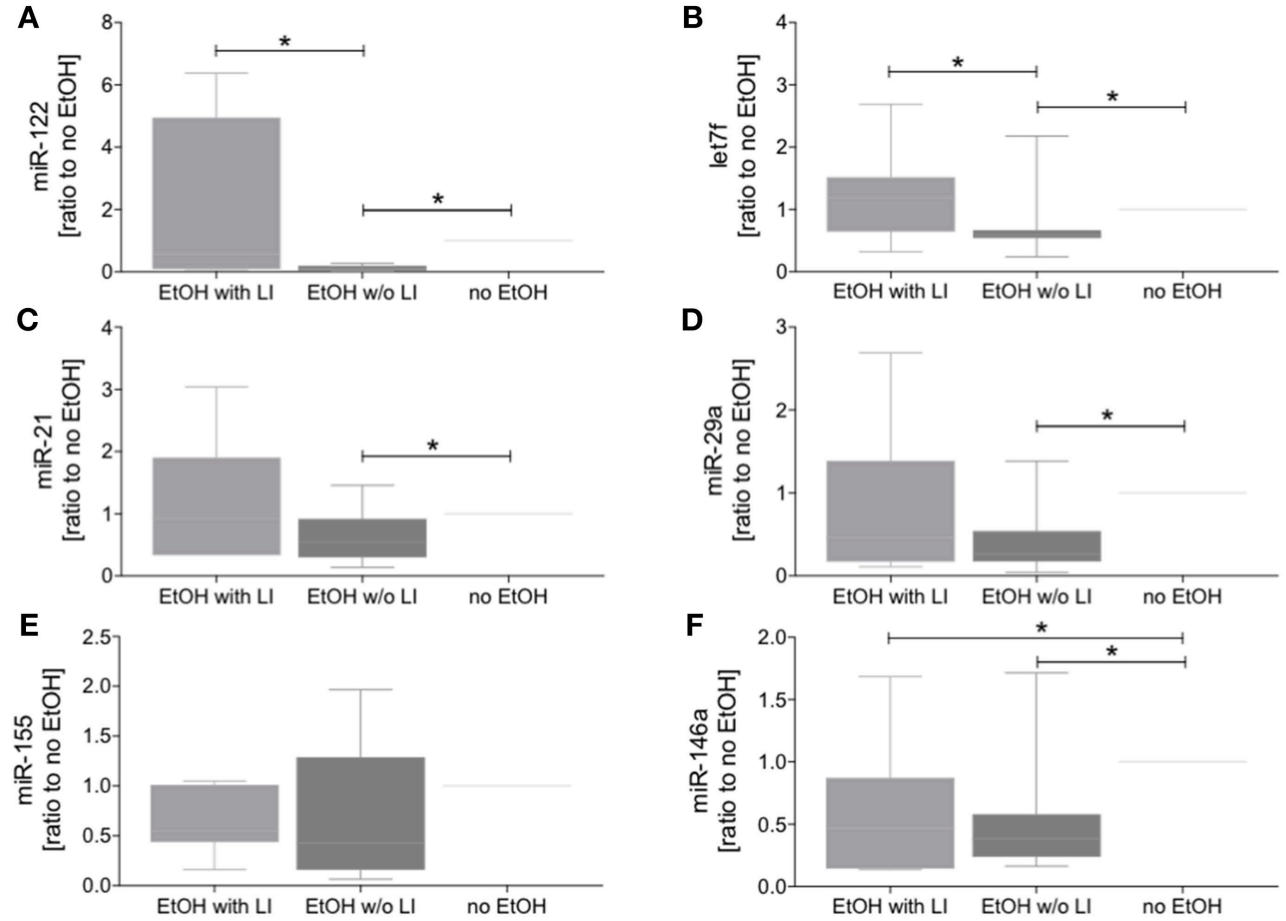
Blood extracellular vesicles (EVs) from alcohol-intoxicated patients have miRNA barcode. The miR level in blood EVs was determined *via* quantitative RT-PCR analysis. Relative expression of miR-122 **(A)**, let7f **(B)**, miR-21 **(C)**, miR-29a **(D)**, miR-155 **(E)**, and miR-146a **(F)** was quantified as ratio to no EtOH. EtOH, ethanol; EtOH with LI, alcohol drinkers with liver injury; EtOH w/o LI, alcohol drinkers without liver injury; no EtOH, non-drinkers. Data are presented as box and whiskers with 5–95 percentile. ^*^p < 0.05.

The findings of the correlation between liver damage and inflammatory changes with EVs led us to further explore EV characteristics. To ascertain whether or not circulating EVs were derived from the liver, we analyzed EV protein composition (47). Circulating EVs were isolated from plasma proteins using a qEV column (Figure 4A) and CD9, a common EV marker (18), which gave us a high level of detection in all groups (Figure 4B). To determine if the EV population present in the blood from alcohol-intoxicated trauma patients could be profiled based on the miR composition, the miR expression pattern of miR-122, let7f, miR-21, miR-29a, miR-155, and miR-146a was analyzed. miR-122 was the most highly expressed miR target within the EtOH with LI group. This increase in miR-122 expression was statistically significant when compared to the no EtOH group (*p* < 0.05, Figure 5A). Interestingly, there was a significant increase in let7f expression within the EtOH with LI group compared to the EtOH w/o LI group (*p* < 0.05, Figure 5B). Further, there was a significant decrease in miR-122, let7f, miR-21, miR-29a, and miR-146a expression patterns when comparing the EtOH w/o LI group *v*ersus the no EtOH group (*p* < 0.05, Figure 5). The expression of miR-146a was significantly lower in the EtOH with LI and EtOH w/o LI groups when compared to the no EtOH group (*p* < 0.05, Figure 5).

### A miR “Barcode” Derived From EVs in Blood From Non-drinkers Can be Used to Identify Alcohol Habituation in Trauma Patients

**Figure 6:**
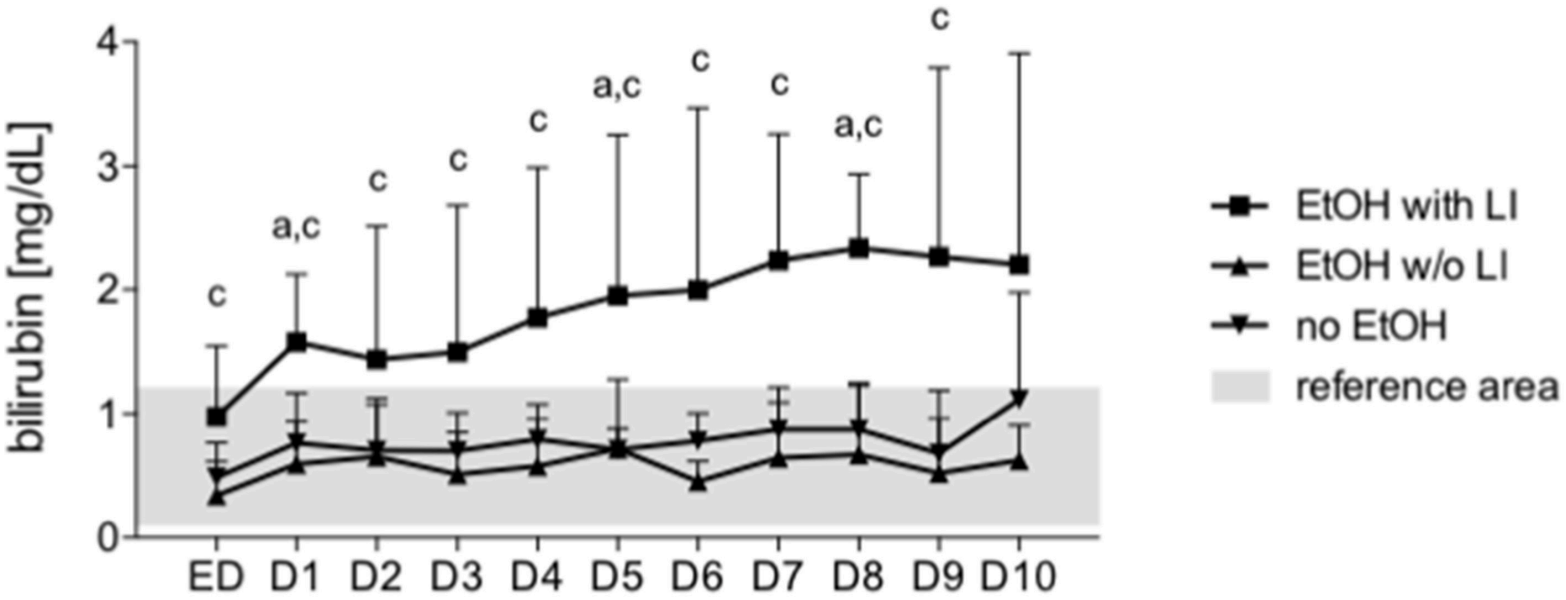
Bilirubin is increased after in trauma patients with liver injury. Serum bilirubin levels were measured every day from the admission to the emergency department (ED) to 10 days after admission daily (1–10). EtOH, ethanol; EtOH with LI, alcohol drinkers with liver injury; EtOH w/o LI, alcohol drinkers without liver injury; no EtOH, non-drinkers. Data are presented as mean ± SD unless stated otherwise. *p* < 0.05 in a: EtOH with LI vs. no EtOH, c: EtOH with LI vs. EtOH w/o LI.

The hypothesis that EVs from trauma patients carrying a specific miR “barcode” can identify patient drinking behavior is supported by the miR profile that is present in EVs released by damaged hepatocytes and is correlated with serum bilirubin. An increase in serum levels of total bilirubin is associated with a reduced capacity for hepatic biosynthesis and marked acute inflammatory reactions, which potentiates (multiple) organ failure and infections (48). We observed in our study that prolonged hyperbilirubinemia, coupled with acute inflammatory reactions and poor platelet count, Figure 6 indicates a high mortality risk for the patient and a fatal outcome with the next occurrence of alcoholic hepatitis (48). There was a significant positive correlation between bilirubin levels and miR-122 expression in EVs at each post-injury day up to day 10, with miR-122 playing a functional role in alcohol intoxication and liver regeneration (Table 4) (25). When compared to nonalcoholics, miR29a expression in the blood EVs from patients with ambulatory alcoholic liver disease was increased and correlated with bilirubin levels up to day 7 in the present study (Table 4) (23, 49). Further, the expression of let7f miR, which is known to be increased in blood EVs from patients with alcoholic liver disease, was increased significantly and correlated with bilirubin levels at day 1, 3, and 4 post trauma episode (Table 4) (23).

**Table 4:** Correlation analyses between miRs and bilirubin.

**Correlation analysis**	**Day**	**Pearson *r***	***p-*value**	**Number of pairs**
miR-122 and bilirubin	ED	**0.7634**	**0.0004**	17
	day 1	**0.7652**	**0.0009**	15
	day 2	**0.8562**	**< 0.0001**	17
	day 3	**0.9228**	**< 0.0001**	16
	day 4	**0.9471**	**< 0.0001**	12
	day 5	**0.8707**	**0.0005**	11
	day 6	**0.9164**	**0.0002**	10
	day 7	**0.8877**	**0.0006**	10
	day 8	**0.8210**	**0.0067**	9
	day 9	**0.9046**	**0.0020**	8
	day 10	**0.8783**	**0.0041**	8
miR-let7f and bilirubin	ED	0.4266	0.0877	17
	day 1	**0.5678**	**0.0272**	15
	day 2	0.4324	0.0830	17
	day 3	**0.5030**	**0.0470**	16
	day 4	**0.6359**	**0.0262**	12
	day 5	0.4930	0.1234	11
	day 6	0.5471	0.1017	10
	day 7	0.3112	0.3814	10
	day 8	0.2759	0.4724	9
	day 9	0.4833	0.2251	8
	day 10	0.4494	0.2640	8
miR-21 and bilirubin	ED	**0.6863**	**0.0023**	17
	day 1	**0.7249**	**0.0022**	15
	day 2	**0.5943**	**0.0119**	17
	day 3	**0.7207**	**0.0016**	16
	day 4	**0.8030**	**0.0017**	12
	day 5	**0.6234**	**0.0404**	11
	day 6	**0.6458**	**0.0437**	10
	day 7	**0.6566**	**0.0392**	10
	day 8	0.5725	0.1072	9
	day 9	0.6945	0.0559	8
	day 10	0.5917	0.1223	8
miR-29a and bilirubin	ED	**0.7332**	**0.0008**	17
	day 1	**0.7092**	**0.0031**	15
	day 2	**0.6909**	**0.0021**	17
	day 3	**0.8195**	**0.0001**	16
	day 4	**0.8349**	**0.0007**	12
	day 5	**0.6775**	**0.0220**	10
	day 6	**0.6768**	**0.0316**	10
	day 7	**0.6528**	**0.0407**	10
	day 8	0.5356	0.1372	9
	day 9	0.6723	0.0678	8
	day 10	0.6258	0.0970	8

*Positive correlation between miRs detected in EVs upon admission of patients to the emergency department (ED) and bilirubin values from ED until day 10 (day 1–10) daily is shown. Bold values indicate significant results*.

## Discussion

Alcohol intoxication, either chronic or acute, is indisputably influencing the development of post-injury complications and outcomes in severely injured trauma patients. However, the overall effect of alcohol remains controversial. Whereas continuous alcohol intoxication promotes inflammation and liver injury, an acute bout of alcohol intoxication, without signs of chronic abuse or liver injury, dampens the inflammatory response (5, 6, 50). Extracellular vesicles (EVs) and microRNA (miR) have emerged as potential biomarkers of disease (23, 51). Here we have shown that EV numbers, along with specific miRs—miR-122 and let7f—were increased in blood EVs from the EtOH with LI group, whereas significantly decreased in the EtOH w/o LI group. These changes were paralleled by either increased inflammation in the EtOH with LI group, or decreased inflammation in the EtOH w/o LI group. Notably, our results suggest that miR-122 significantly correlates with liver injury, which possibly occurred due to continuous alcohol intoxication. Our novel data in trauma patients demonstrate that hepatocyte damage in alcoholic liver injury may be reflected by enhanced EVs containing a specific miR “barcode” and the correlated increase of the systemic inflammatory marker IL-6. Furthermore, increased plasma levels of IL-33, which were detected in the EtOH with LI group, confirm that this group harbors ongoing liver injury, which is in line with a previous study that found IL-33 was not significantly elevated in heavy drinkers when compared to healthy individuals, but was markedly increased in ALD patients (38).

In our study, following a recently reported finding by Scheyerer et al., we found no significant relationship between alcohol intoxication and injury severity (52). There was, however, a trend toward a decrease in ISS within the EtOH w/o LI group, which has been shown by others as well (53). With regard to the type of injury, there was a clear trend toward a higher prevalence of head trauma in alcohol-intoxicated patients, which is also in line with other studies (54, 55). Current clinical studies have demonstrated that alcohol intoxication has been associated with decreased rates of pneumonia, or even improved mortality rates, after trauma or hemorrhagic shock *in vivo* (11, 12, 14). However, other findings highlight that alcohol did not increase the incidence of sepsis, other post-injury complications, or mortality (10, 56, 57). Interestingly, there is also evidence that alcohol intoxication elevated the risk for in-hospital complications (6). In our study, the incidence of pneumonia was highest within the EtOH with LI group. However, we did not find significant changes in the development of sepsis or septic shock. Similarly, no association between mortality rates and alcohol intoxication have been noted elsewhere in the literature. These intriguing findings are supported by *in vivo* mouse studies showing that acute alcohol-intoxication may be responsible for improved clinical outcomes via decreased neurologic and inflammatory impacts typically observed in traumatic brain injury (58). Anti-inflammatory properties of acute alcohol intoxication, which are linked to potentially beneficial outcomes, have been associated with decreased levels of IL-6 (12). Clinical data and results from this study support the findings that the initiation of an overwhelming inflammatory response after trauma is lowered in alcohol-intoxicated patients who do not show signs of liver injury, as reflected by the reduced systemic IL-6 values and leukocyte counts upon hospital admission (59, 60). On the one hand, it is known that leukocytes play a decisive role in post-traumatic immunity and in the initiation of the inflammatory cascade *via* secretion of pro-inflammatory cytokines including IL-6 (61, 62), and in a previous study we found a significant correlation between decreased leukocyte counts and reduced IL-6 levels upon hospital admission. In that study, we surmised that the primary determinant of reduced systemic IL-6 levels was probably a decrease in total leukocytes, which was influenced by patient BAC levels. In this study we focused on IL-6 due to its established use in the clinical setting, its apparent involvement in alcohol-modified inflammation and it is indicative of leukocyte activation and ongoing inflammation. Additionally, the analysis of other cytokines, including IL-1beta, TNF-alpha, and IL-10 did not provide any significant differences between the groups. Continuous alcohol intoxication, which is paralleled by marked inflammation, has been linked to more than 60 chronic medical conditions (63). Recently, a close correlation between cell death markers and markers of systemic inflammation, hepatic failure, ALT and bilirubin was observed, but not with markers of extra-hepatic organ injury in acute and chronic liver failure (64). With regard to more specific traumatic injuries, impaired outcomes resulted more frequently in patients presenting with liver cirrhosis (5). Thus, a pre-existing condition, such as cirrhosis, should be included in trauma scores when assessing an individual's mortality risk profile. Current biomarkers, including GGT, AST, and ALT can provide information regarding historical patterns of alcohol consumption and the degree of alcoholic liver injury, but these assays are limited by low sensitivity and specificity (63). Given these limitations, most causes of liver cell injury are associated with a greater increase in ALT over AST; however, an AST to ALT ratio of 2:1 or greater is suggestive of ALD, particularly in the setting of an elevated GGT (65). Our study found that a significantly increased AST:ALT ratio, paralleled by significantly elevated GGT levels, strongly suggests continuous, and most likely, chronic alcohol consumption. This is notable, seeing as how all patients with other chronic illness, including patients with viral hepatitis, were excluded from our study. Unfortunately, we have yet to perform the self-report Alcohol Use Disorders Identification Test (AUDIT), which in most cases will provide more accurate results. However, within the trauma setting of our study, patients are usually unable to present an accurate drinking history. Therefore, we are unable to provide any data regarding patient drinking behavior or historical patterns.

In order to provide evidence that the EtOH with LI group arrived at the emergency room afflicted with latent liver injury we analyzed serum IL-33 (as a DAMP), or alarmin levels. Recently, it has been shown that systemic levels of IL-33 are not elevated in heavy drinkers when compared to healthy individuals, but IL-33 is markedly increased in ALD patients (38). If we observed elevated levels of IL-33 in our group of suspected chronic EtOH drinkers, we therefore assumed an ongoing liver injury.

Prompt recognition of historical alcohol abuse included in an overall liver injury assessment can be life-saving. One of the key factors limiting the development of an effective therapy is the lack of non-invasive and reliable biomarkers that may help identify patients at risk of developing post-traumatic complications.

Recent studies report that the number of circulating EVs increases in murine models of nonalcoholic liver disease and ALD (22, 24). *In vitro* ethanol exposure of hepato-derived Huh7.5 or HepG2 cells confirmed the release of EVs (24, 66). Additionally, during the development of alcoholic steatohepatitis, hepatocyte-specific EVs expressing a miR “barcode” can be measured in the circulation (23). The current study builds on these findings and demonstrates that the amount of circulating EVs is increased in the EtOH with LI group. Moreover, the amount of EVs correlated positively with ALT and IL-6, thus confirming the aforementioned inflammatory changes that are mechanistically associated with liver damage. Furthermore, two miRs were increased in blood EV samples from the EtOH with LI group: miR-122 and let7f. Post trauma, miR-21 and miR-29a were not significantly increased in the EtOH with LI group when compared with the EtOH w/o LI group, though there appears to be a trend in the data. Interestingly, five miRs from blood EVs—miR-122, let7f, miR-21, miR-29a, and miR-146a—showed decreased expression in the EtOH w/o LI group. Previous reports suggest that blood EVs and let7f, as well as miR-29, are increased in ALD patients with active alcohol consumption compared to nonalcoholics (23), further supporting our findings in trauma patients. Other miRs with known functional roles in metabolism and hepatic homeostasis are altered by chronic ethanol feeding (adaptation) as well. Notably, the expression of liver-specific miR-122 is significantly increased during ethanol adaptation (25). In the present study, miR-122 was the miR most tightly correlated with bilirubin levels. miR-21 was closely associated with fibrotic liver disease *in vivo*, and therefore proposed as a potential plasma biomarker for hepatic fibrosis (26). Interestingly, miR-146a levels were significantly decreased in both EtOH groups, which elucidates an intriguing hypothesis that must be explored further in future studies. It has been shown that miR-146a contributes to the negative regulation of pro-inflammatory cytokine secretion, and that maintaining 146a homeostasis plays a key role in controlling the inflammatory response (27). miR-146a has also been shown to play an important role in the resolution of the pro-inflammatory reaction. miR-146a can respond to pro-inflammatory stimuli such as IL-1beta or endotoxin, leading to the inhibition of the inflammatory response in macrophages, monocytes, and keratinocytes (28, 29). Consequently, miR-146a is significantly involved in the regulation of inflammation. Decreased levels of miR-146a in EVs from alcohol-intoxicated trauma patients with liver injury reveals immune dysregulation and ongoing inflammation in those patients. In the post-injury phase, trauma frequently results in inflammatory complications that affect immune system homeostasis, which, in some cases, leads to sepsis, septic shock, or multiple organ dysfunction syndrome (MODS) (67). The development of these complications is caused by the dysregulation of the compensatory anti-inflammatory response in trauma that should simultaneously equalize the pro-inflammatory response to avoid tissue damage and excessive cytokine release (67). Therefore, the miRs described in this study, particularly miR-146a, require further analysis because they possess biomarker capabilities and play a potential regulatory role.

There are several limitations embedded within the present study. We have not generated a miR profile on circulating EVs *via* miR-sequencing. It is also important that we embark on a large-scale study in the future to evaluate IL-33, as it has been shown to be upregulated in patients suffering from alcoholic liver disease. We were only able to build our cohort from a very small number of patients with liver injury caused by chronic alcoholism. Our study related to specific EV “barcodes,” and using IL-33 as an indicator of alcohol-related liver injury, requires further observations using a larger cohort of trauma patients with or without liver injury due to alcohol abuse or other causes of liver injury. Our group also needs to perform a comparative analysis between EV miR profile and IL-33 specificity and/or sensitivity as an indicator of liver disease in alcohol-intoxicated traumatized patients. It remains an open determination whether or not there is a need for expensive and extensive miR analysis when IL-33 quantification may provide comparable specificity and sensitivity. Additionally, future studies should include AUDIT scores to assess patient drinking behavior and patterns. Seeing as how none of the applied biomarkers currently offer perfect validity reflecting heavy drinking and associated liver injury, additional biomarkers such as carbohydrate-deficient transferrin or some ethanol-specific metabolites (e.g., ethyl glucuronide, phosphatidylethanol, protein-acetaldehyde) would provide helpful information for diagnostic purposes.

Patients presenting with documented chronic liver illness, such as hepatitis B or C infection, were excluded in this study. However, we were unable to further diagnose the etiology of LI within our cohort beyond the exclusion parameters, especially in no EtOH group.

In summary, alcohol affects EVs and their miR “barcode.” Our study suggests that EVs and their specific miR “barcode” may have the potential to divulge pre-traumatic liver injury in severely injured, alcohol-intoxicated trauma patients. Interestingly, the correlation between IL-6 levels and EVs, with subsequent alcohol-mediated changes and inherent “barcode” dynamics, may highlight a greater role in regulating immunological processes upon alcohol intoxication and traumatic insult. Deeper insight into miR regulation of specific gene expression, and into the transcriptional regulation of miR expression overall, is required to obtain a better understanding of how pre-traumatic liver injuries will affect the immune response to trauma.

## Author Contributions

BR designed the study, performed the statistical analysis and wrote the first draft of the manuscript. AE designed the study, carried out the analyses, and contributed to the intellectual content of the manuscript and revised it. NF collected the clinical data and carried out the analyses. NW, YK, MI, FH, YT, and IM revised the manuscript and made important intellectual contributions to the completion of the study.

### Conflict of Interest Statement

The authors declare that the research was conducted in the absence of any commercial or financial relationships that could be construed as a potential conflict of interest.
